# Targeting Spatiotemporal Heterogeneity of oncomiRNAs: A New Frontier in Cancer Therapy

**DOI:** 10.1002/cnr2.70639

**Published:** 2026-08-09

**Authors:** T. Jeethy Ram, Muralee Damodaran

**Affiliations:** ^1^ Department of Health Sciences Research Amrita Institute of Medical Sciences, Amrita Vishwa Vidyapeetham Kochi Kerala India

**Keywords:** cancer progression, liquid biopsy, microRNA biomarkers, oncomiRNA, spatiotemporal heterogeneity, spatiotemporal miRNA profiling

## Abstract

**Background:**

miRNAs are short RNA transcripts that modulate gene expression after transcription and have emerged as pivotal regulators of cancer biology. A subset, termed oncomiRNAs, functions as oncogenes or tumor suppressors, influencing key cellular events such as cell growth, programmed cell death, neovascularization, tissue invasion, and metastatic spread. Dysregulation of these miRNAs drives tumor initiation and progression, underscoring their role in cancer evolution. Traditionally, studies have relied on bulk tissue analyses, overlooking the profound spatiotemporal heterogeneity of oncomiRNA expression, including variations across tumor regions, metastatic sites, disease stages, and during treatment.

**Recent Findings:**

Advances in spatial transcriptomics, single‐cell profiling, and longitudinal liquid biopsy technologies have provided new insights into the dynamic regulation of oncomiRNAs. These approaches reveal significant spatiotemporal variability in miRNA expression and are increasingly implicated in shaping tumor heterogeneity, therapeutic resistance, immune evasion, and divergent clinical outcomes. Emerging evidence underscores the importance of integrating these dynamic molecular patterns into biomarker discovery frameworks and precision oncology strategies. Furthermore, the incorporation of artificial intelligence and multi‐omics data integration is enhancing patient stratification and predictive modeling.

**Conclusion:**

A comprehensive understanding of the spatiotemporal regulation of oncomiRNAs is essential for advancing next‐generation cancer diagnostics and therapeutics. Future efforts should focus on systematic multi‐region and longitudinal study designs, as well as their integration into adaptive clinical trials. Leveraging these insights may enable miRNA‐guided, stage‐adapted precision cancer theranostics in heterogeneous malignancies.

## 1 Introduction

miRNAs are non‐coding RNAs, 20–22 nucleotides in length, that act as key regulators of gene expression and are evolutionarily conserved across species [1, 2]. They execute this modulation by binding to 3′UTR (3‐prime untranslated region) with complementary sequences in target gene transcripts, leading to translational repression or mRNA degradation [2]. miRNAs have emerged as major regulators in cancer biology due to their ability to orchestrate various cellular mechanisms such as cell division, maturation, angiogenesis, apoptosis, invasion, and metastasis [3]. As of now, over 1900 pre‐miRNA sequences have been annotated in the human genome, highlighting their crucial significance in the regulatory framework of the genome [4]. A subset known as oncomiRNAs operates as either oncogenes or tumor suppressors, contingent upon the cellular and molecular context. Mounting evidence indicates that miRNA dysregulation may facilitate tumorigenesis by influencing several key oncogenic pathways. These include pathways governing cell cycle, p53 signaling, Wnt/β‐catenin, NF‐kappaB, phosphoinositide 3‐kinase/AKT, and Hh (hedgehog) signaling cascades thereby promoting malignant transformation, sustaining cellular proliferation, enabling metastatic behavior, and conferring resistance to therapy [3, 5, 6, 7]. In parallel, the concepts of cells of origin and cancer stem cells (CSCs) have shed light on the hierarchical organization of tumors, wherein cells of origin initiate malignancy and CSCs maintain tumor growth and recurrence [8, 9]. The dysregulation of specific miRNAs has been implicated in the maintenance and behavior of these subpopulations, positioning miRNAs as promising targets for both diagnostic and therapeutic strategies in cancer management [10]. The small size, stability, and high specificity of miRNAs allow them to function as both biomarkers and therapeutic agents [11]. Advances in nanotechnology and chemical biology have enabled the development of bioanalytical tools and miRNA‐modulating agents, such as small molecules and synthetic oligonucleotides, to manipulate miRNA expression or function. Additionally, high‐throughput screening methods have facilitated the discovery of small molecules capable of modulating endogenous miRNA biogenesis [12]. These developments highlight novel, precise, and effective options for combating miRNA‐associated malignancies.

Historically, research on oncomiRNAs has depended on bulk tissue profiling, which presumes homogeneity among tumors and disease phases. However, cancer is inherently diverse, with variability observed across tumor regions and their microenvironment (spatial heterogeneity), as well as through different stages of its development, from initiation to recurrence (temporal heterogeneity) [13]. Emerging evidence suggests that oncomiRNA expression and function are dynamic and exhibit significant spatiotemporal heterogeneity, likely influenced by factors such as regional tumor architecture, hypoxic gradients, immune contexture, and disease stage [14, 15, 16]. Spatially, differential miRNA expression has been observed across tumor subregions. For example, Alfardus et al. reported distinct miRNA expression patterns between the tumor core/rim and invasive margin in glioblastoma, highlighting region‐specific heterogeneity associated with metabolic adaptation and tumor behavior [17]. Such spatial variation likely reflects localized differences in cellular composition, including cancer‐associated fibroblasts (CAFs), tumor associated macrophages (TAMs), and infiltrating immune cells within the tumor microenvironment (TME).

Temporally, miRNA expression evolves across disease progression. For example, miR‐10b has been associated with advanced tumor stage, lymph node and distant metastasis, and poor prognosis in colorectal [18, 19], gastric [20], and melanoma [21] cancers, suggesting its role in tumor progression. However, these findings are largely correlative and based on bulk tumor or circulating measurements, and thus are more indicative of biomarker potential than direct mechanistic causality. Conversely, studies in small cell carcinoma of cervix have shown downregulation of miR‐10b along with other miRNAs (miR‐143, miR‐199a, miR‐125b, let‐7c, miR‐145, and miR‐100) in advanced‐stage tissues compared to early‐stage tissues [22], highlighting the context‐dependent and stage‐specific variation in miRNA expression during cancer progression.

Importantly, a distinction should be made between tumor tissue‐derived miRNA data, which can provide mechanistic insights, and circulating miRNAs (serum, plasma, or exosomal), which primarily reflect tumor burden and systemic responses. While circulating miRNAs hold strong promise as minimally invasive biomarkers, their dynamic changes do not necessarily imply direct functional roles within tumor cells without experimental validation.

Despite advances in spatial transcriptomics, single‐cell profiling, and longitudinal liquid biopsy technologies, the field still lacks a unified mechanistic framework linking spatiotemporal miRNA dynamics to cancer progression. Spatial gradients in oxygenation and stromal composition can drive region‐specific activation of pathways such as HIF‐1α signaling, TGF‐β/EMT, and PI3K/AKT, mediated in part by localized miRNA expression. Concurrently, temporal shifts in oncomiRNA profiles may contribute to the emergence of drug‐tolerant persister cells, facilitate immune evasion, and support metastatic dissemination. Thus, spatiotemporal variation in oncomiRNA expression is increasingly recognized as a functional component of tumor evolution and treatment resistance.

Advances in single‐cell and spatial miRNA profiling, together with longitudinal liquid biopsy approaches, have deepened our understanding of oncomiRNA heterogeneity across time and space, and spurred investigations into their use as biomarkers and therapeutic targets. Nevertheless, a structured narrative survey of the spatiotemporal regulation of oncomiRNAs remains limited and a deeper understanding of these dynamic regulatory networks is essential for improving precision oncology approaches.

In this review, we highlight the emerging significance of spatiotemporal oncomiRNA regulation and discuss how integrating these dynamics into biomarker discovery, therapeutic targeting, and adaptive clinical strategies may enhance cancer diagnosis, prognostication, and treatment outcomes. Critically, this review is distinguished from standard “miRNA in cancer” reviews by its specific focus on three interrelated dimensions: (i) spatial gradients in oncomiRNA expression across tumor subregions, microenvironmental niches, and metastatic sites; (ii) temporal trajectories of miRNA dynamics during disease progression and in response to therapy; and (iii) cross‐compartment discordance between tissue‐derived and circulating miRNA profiles. Integrating these dimensions reshapes both (a) biomarker design, by moving beyond single‐timepoint bulk profiling toward longitudinal, multi‐region, and liquid biopsy‐integrated strategies, and (b) therapeutic targeting, by enabling stage‐adapted, spatially informed intervention frameworks. The literature included in this review was surveyed as a structured narrative up to April 2026, ensuring representation of recent advances in spatiotemporal miRNA research. miRNA nomenclature is presented as reported in the original studies; strand‐specific annotations are included only where specified.

### 1.1 OncomiRNAs and Their Role in Cancer Progression

The involvement of oncomiRNAs in cancer has been extensively studied. These microRNAs, when abnormally overexpressed, contribute significantly to tumor initiation and progression. Increased oncomiRNA levels are commonly observed in tumor cells and in the surrounding tumor microenvironment, where they facilitate carcinogenic processes like uncontrolled cell proliferation, migration, invasion, and metastasis. OncomiRNAs typically operate by inhibiting tumor suppressor (TS) genes and may also hinder the immune system's capacity to produce a potent anti‐tumor response. Prominent oncomiRNAs, including miR‐17‐92 cluster‐associated miRNAs and others involved in metastasis, inflammation, and proliferation, have been widely studied for their contributions to cancer development.

For instance, in pancreatic ductal adenocarcinoma (PDAC), elevated circulating miR‐10b levels have been reported. In parallel, functional studies demonstrate that miR‐10b promotes tumor growth, migration, epithelial–mesenchymal transition (EMT), and invasion, partly through direct suppression of TIP30, resulting in enhanced EGFR signaling and amplification of EGF‐driven pathways. This signaling cascade enables cross‐talk with TGF‐β, promoting EMT and invasive behavior, alongside downregulation of metastasis‐suppressive genes such as RAP2A, EPHB2, KLF4, and NF1 [23].

Therapeutically, inhibition of miR‐10b using the targeted nanodrug MN‐anti‐miR‐10b has demonstrated near‐complete in vivo knockdown (~99%) and suppression of metastatic traits. These effects are associated with reduced stem cell–like properties and support a dedifferentiation‐based mechanism, whereby metastasis‐initiating, stem‐like cancer cells are preferentially targeted and driven toward a less aggressive phenotype [24].

The functional role of miR‐10 family members is highly pleiotropic and context‐dependent, varying across tumor types. In hepatocellular carcinoma (HCC), miR‐10a‐5p is downregulated in aggressive tumor phenotypes, including those associated with microvascular invasion (MVI), and exerts tumor‐suppressive effects. Mechanistically, miR‐10a‐5p directly targets TFR1, leading to reduced CD24 expression and inhibition of STAT3 signaling, thereby suppressing cell proliferation and promoting apoptosis [25]. Similarly, in gastric cancer, miR‐10b‐5p exhibits tumor‐suppressive activity by directly targeting Tiam1, leading to inhibition of cell proliferation and migration, along with reduced tumor growth and increased apoptosis in xenograft models [26]. In contrast, in glioblastoma, miR‐10b is upregulated and correlates with higher tumor grade, with expression levels significantly associated with invasion‐related mediators such as RhoC and uPAR at both mRNA and protein levels (*p* < 0.05), suggesting a role in invasive phenotypes [27]. In breast cancer, miR‐10b promotes invasion and metastasis through repression of HOXD10, leading to upregulation of RHOC. Its expression is transcriptionally induced by TWIST, and functional studies demonstrate that miR‐10b overexpression is sufficient to initiate invasion and metastasis in vivo [28]. In cervical cancer, extracellular vesicle‐associated miR‐10a‐5p derived from cancer‐associated fibroblasts enhances angiogenesis and tumor growth via TBX5‐mediated activation of the Hedgehog pathway, highlighting a microenvironment‐driven mechanism [29]. These observations highlight the context‐dependent roles of oncomiRNAs across different tumor types.

miR‐21 is one of the most consistently upregulated oncomiRNAs across multiple solid tumors [30]. In colorectal cancer (CRC), inflammatory COX‐2/PGE2 signaling has been shown to upregulate miR‐21 expression, leading to suppression of the tumor suppressor PDCD4 and promoting disease progression [31]. Preclinical studies demonstrate that STAT3‐driven miR‐21 promotes tumor progression via PTEN suppression, with antagomir‐mediated inhibition reducing tumor burden in vivo; elevated miR‐21 expression also correlates with poor patient survival [32]. In addition to these mechanistic roles, miR‐21 has been associated with cancer stem cell–related phenotypes in prostate cancer, based on combined experimental and clinical observations [33]. However, these findings remain largely associative and require further mechanistic and clinical validation to establish their functional and translational relevance.

miR‐155 is a well‐characterized oncomiRNA with context‐dependent roles, frequently upregulated across multiple malignancies, including breast, lung, pancreatic, and colorectal cancers, as well as lymphomas [34], while also contributing to epithelial homeostasis under physiological conditions [35].

In breast cancer, mechanistic studies demonstrate that miR‐155 promotes proliferation, migration, and invasion through modulation of multiple oncogenic pathways. Notably, miR‐155 enhances tumor cell proliferation and migration via downregulation of SOCS1 and upregulation of MMP16 [36]. Inhibition of miR‐155 induces apoptotic cell death and cell cycle arrest, accompanied by increased expression of pro‐apoptotic factors (BAX, cytochrome c, caspases 3/8/9) and reduced BCL‐2 levels, along with suppression of cell migration and invasion [37]. Bioinformatic and expression analyses from the same study further suggest MAPK7 as a potential downstream target of miR‐155, although direct regulation requires further validation [37].

Beyond these roles, miR‐155 has been implicated in the regulation of cancer stem‐like properties, where its overexpression enhances tumor sphere formation and stemness‐associated markers (CD44, CD90, ABCG2), while its inhibition increases CD24 expression and sensitizes cells to chemotherapeutic agents such as doxorubicin, supporting a role in therapeutic resistance and tumor recurrence [38].

Notably, regulatory interactions such as the FOXP3/miR‐155 axis also suggest a role in maintaining epithelial homeostasis through modulation of ZEB2 [35], underscoring the dual context‐dependent roles of miR‐155 in both normal physiology and cancer.

Collectively, while substantial mechanistic evidence supports the oncogenic functions of miR‐155, its context‐dependent roles and involvement in multiple regulatory networks necessitate careful interpretation, particularly when translating these findings to clinical settings.

miR‐17‐5p, a prominent component of the oncogenic miR‐17‐92 cluster, is implicated in tumorigenesis and progression across multiple malignancies, including liver, gastric, colorectal, prostate, breast, and lung cancers [39]. In clinical settings, circulating miR‐17‐5p and miR‐20a levels have been associated with advanced disease features and poorer survival outcomes in gastric cancer. Notably, miR‐20a emerged as an independent prognostic marker in multivariate analysis (HR 1.58, 95% CI 1.10–2.25, *p* = 0.013), whereas miR‐17‐5p, despite showing significant associations with disease progression (HR 1.79, 95% CI 1.11–2.87, *p* = 0.017), did not retain independent prognostic significance [40]. Complementing these findings, serum exosomal members of the miR‐17‐92 cluster are elevated in gastric cancer and correlate with tumor size, invasion depth, lymph node involvement, distant metastasis, and TNM stage. Diagnostic evaluation demonstrated moderate performance of individual miRNAs (AUC 0.69–0.75), while a combined exosomal miR‐17‐92 panel improved accuracy (AUC 0.81, sensitivity 90.3%, specificity 70%), outperforming individual markers [41]. Collectively, these observations highlight their utility as non‐invasive biomarkers for disease progression. However, circulating miRNA levels primarily reflect systemic tumor dynamics and may not directly represent intracellular functional activity.

In contrast, mechanistic studies in tumor models demonstrate that miR‐17‐5p directly regulates oncogenic pathways. In laryngeal squamous cell carcinoma (LSCC), silencing miR‐17‐5p suppresses proliferation and induces apoptosis, mediated in part through direct targeting of PIK3R1 and modulation of PI3K/AKT signaling, with downstream effects on apoptotic regulators including BCL‐2, BAX, and caspase‐3 [42]. Similarly, in lung adenocarcinoma, miR‐17‐5p is upregulated and promotes proliferation, migration, and invasion while inhibiting apoptosis, partly through direct targeting of SIK1 [43]. Together, these findings underscore the dual role of miR‐17‐5p as a circulating biomarker and a functional regulator of oncogenic signaling, with context‐dependent contributions across tumor types.

While many studies report strong associations with clinical and pathological features, only a subset provides direct mechanistic validation through functional assays. Careful interpretation is therefore required to distinguish biomarker potential from causal functional contribution. This highlights the need for integrative studies addressing the spatial and temporal dynamics of oncomiRNA expression and their roles in tumor evolution and disease progression.

## 2 Spatiotemporal Heterogeneity of oncomiRNAs

**FIGURE 1:**
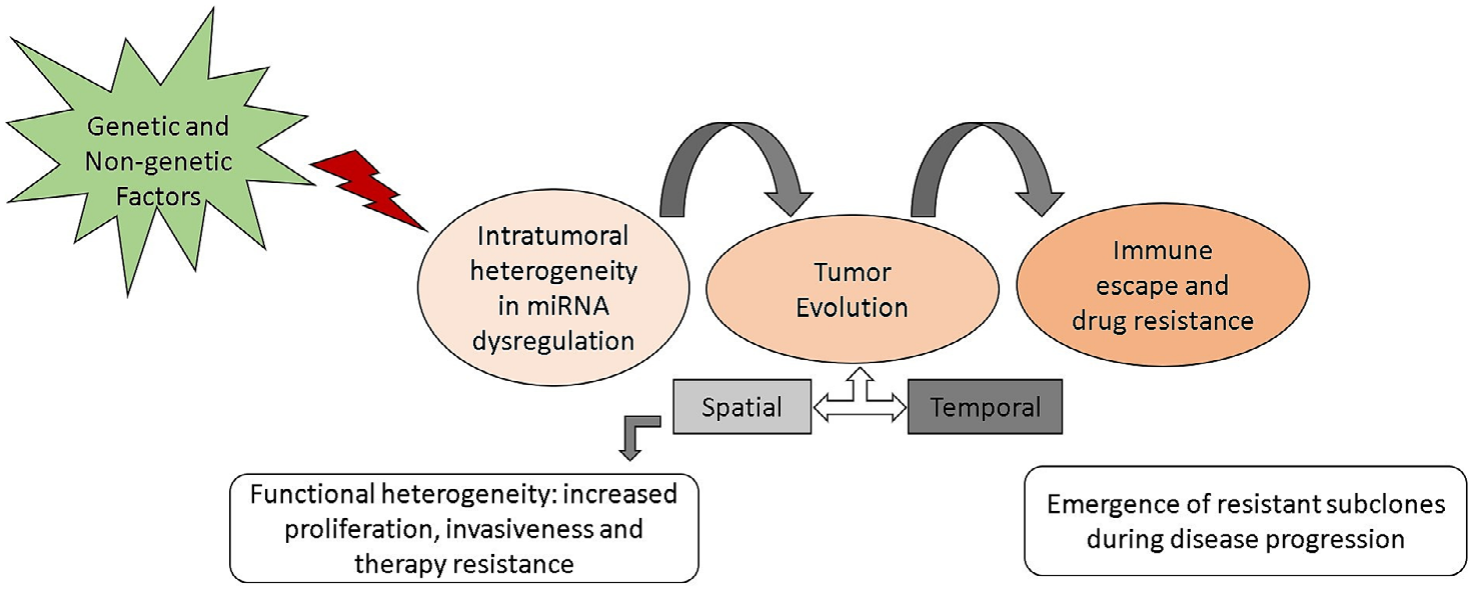
Intratumoral heterogeneity in miRNA dysregulation (altered expression, mutations, and changes in miRNA processing) drives tumor evolution and contributes to drug resistance and immune escape through spatial and temporal dynamics. This figure was created by the authors and has not been published previously.

The term “tumor heterogeneity” encompasses spatial and temporal variations within a tumor (intra‐tumor heterogeneity) as well as differences between tumor sites within the same patient or across patients (inter‐tumor heterogeneity). This heterogeneity arises from genetic and non‐genetic factors, including epigenetic instability, alterations in the tumor microenvironment, and selective pressures from therapy. miRNAs exhibit dynamic, stage‐specific expression patterns that evolve with disease progression, both reflecting and contributing to this heterogeneity (Figure 1).

Spatiotemporal heterogeneity in oncomiRNA expression is not merely descriptive but functionally consequential. Spatial gradients in hypoxia, stromal composition, and immune infiltration are known to influence localized miRNA programs that modulate key signaling pathways such as HIF‐1α, TGF‐β/EMT, and PI3K/AKT [44, 45, 46]. These region‐specific miRNA networks promote phenotypic diversification, including invasive behavior at tumor margins and immune evasion within immunosuppressive niches. Temporally, dynamic shifts in oncomiRNA expression during disease progression and therapeutic exposure have been implicated in adaptive resistance, including the emergence of drug‐tolerant persister cells [47, 48], maintenance of cancer stem‐like populations, and adaptation to selective pressures. Collectively, these processes establish a mechanistic link between spatiotemporal miRNA variation and tumor evolution, ultimately contributing to metastasis, therapeutic resistance, and disease recurrence.

Profiling miRNA expression enables capture of baseline tumor heterogeneity and tracking of adaptive changes in tumor cell populations that drive therapeutic resistance. Moreover, miRNA profiling integrates multiple oncogenic signals beyond the constraints of purely genetic or phenotypic analyses, providing a more comprehensive and integrative view of tumor biology.

The ability to assess miRNAs through minimally invasive liquid biopsies has further advanced the spatiotemporal profiling of circulating miRNAs, establishing liquid biopsy‐based approaches as valuable strategies to monitor tumor dynamics and evaluate therapeutic responses. However, it is important to distinguish between tumor‐derived miRNA expression, which may provide mechanistic insight, and circulating miRNAs, which primarily reflect systemic tumor burden, host response, and intercellular communication. This distinction is critical for accurate interpretation and may inform clinical decision‐making in the context of heterogeneous malignancies.

### 2.1 Spatial Heterogeneity of oncomiRNAs

Cancer progression is often associated with systemic dissemination, characterized by the presence of circulating tumor cells and micro‐ and macrometastases. Thus, when considering intra‐tumor phenotypic heterogeneity, it is insufficient to focus solely on the primary tumor. This is particularly important because distant metastases account for the majority of cancer‐related deaths, yet treatment decisions are typically guided by analyses of the primary tumor alone. Differences in molecular and phenotypic characteristics between primary and metastatic sites can therefore lead to misdiagnosis or suboptimal therapy. Indeed, numerous studies have documented discrepancies in diagnostic markers between primary tumors and their metastases [49]. Given the profound clinical implications of such discordance, a deeper understanding of how primary lesions differ from metastatic lesions is essential for improving patient management. However, experimental data addressing these differences remain relatively limited. This complexity extends to the expression of oncomiRNAs themselves, which can vary markedly across different tumor regions and metastatic sites. Region‐specific miRNA profiling in breast cancer demonstrates substantial intratumoral heterogeneity, with significant variability in miRNA expression observed across multiple sampled regions (central, intermediate, and peripheral) as well as among matched lymph node metastases from the same patient. Despite high technical reproducibility in RNA extraction and RT‐qPCR, miRNA expression exhibited coefficients of variation of up to ~40% within both primary tumors and metastatic sites. Importantly, this variability was observed across all sampled regions, highlighting the impact of sampling bias and the absence of uniform miRNA expression patterns within tumors. These findings emphasize the spatial complexity of oncomiRNA distribution and underscore the need for multi‐region sampling to accurately characterize miRNA expression profiles in primary and metastatic breast cancer [50]. It should be noted, as a general caution, that circulating miRNA changes can reflect tumor burden and systemic inflammation rather than direct mechanistic activity; they should not be interpreted as functionally causal unless paired with intratumoral target engagement data or functional perturbation experiments.

**FIGURE 2:**
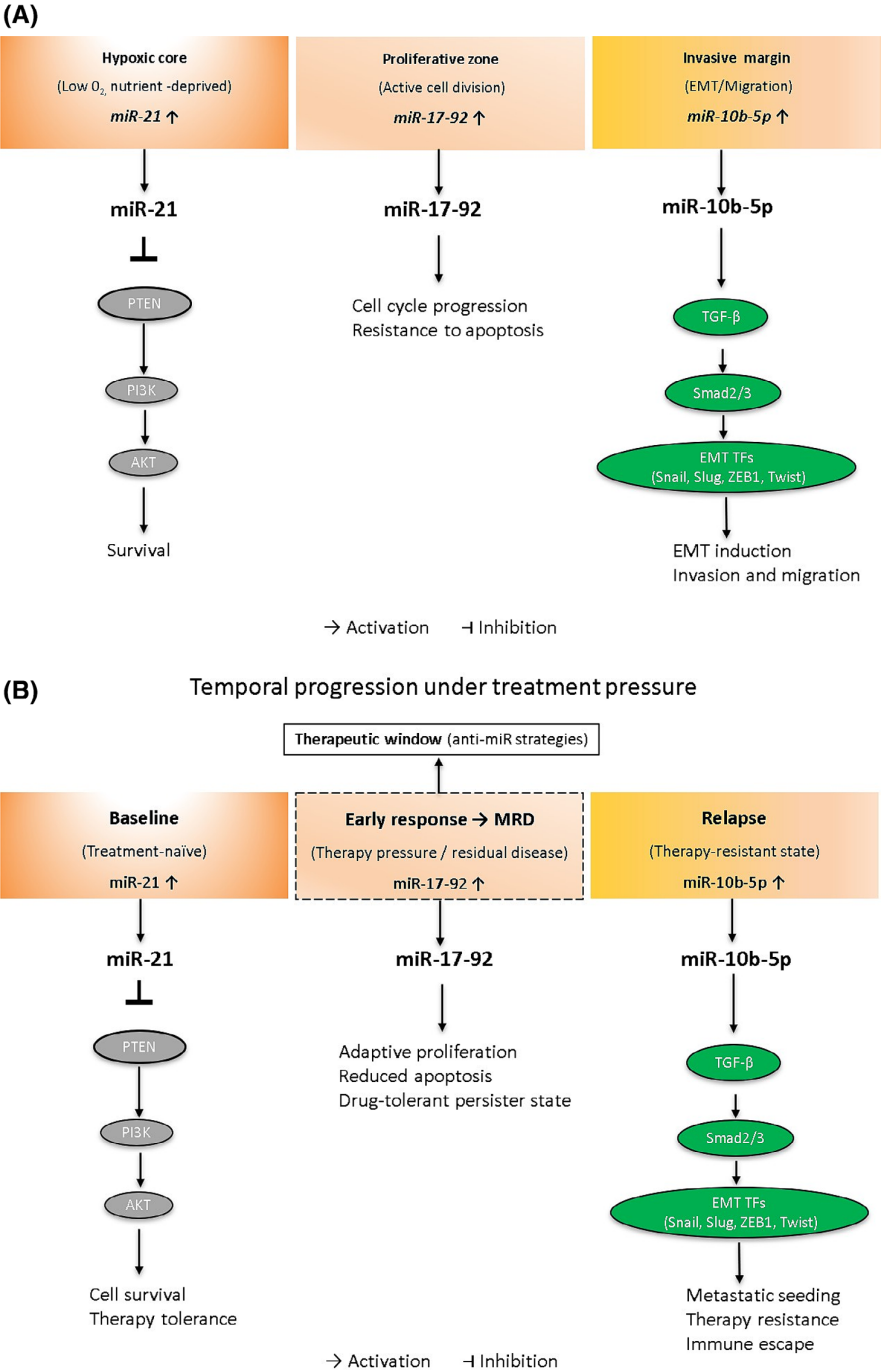
Conceptual framework of spatiotemporal oncomiRNA regulation in cancer. (A) Spatial heterogeneity: distinct tumor regions (hypoxic core, proliferative zone, invasive margin) exhibit differential expression of oncomiRNAs (e.g., miR‐21, miR‐17‐92, miR‐10b‐5p), modulating key pathways including PTEN/PI3K/AKT, and TGF‐ β/EMT signaling. (B) Temporal heterogeneity: dynamic changes in oncomiRNA expression occur across treatment phases (baseline → early response → minimal residual disease → relapse). miR‐21 supports early survival signaling, while therapy‐induced stress elevates miR‐17‐92, promoting adaptive proliferation and drug‐tolerant persister formation during minimal residual disease (MRD). At relapse, miR‐10b‐5p drives EMT‐associated metastatic seeding, therapy resistance, and immune escape. These dynamics highlight critical windows for therapeutic intervention, including anti‐miR strategies during MRD. This figure was created by the authors and has not been published previously.

Consistent with these findings, spatial heterogeneity of miRNA expression has also been reported in colorectal cancer. The localized upregulation of miR‐17‐3p and miR‐92a at the invasive front of pT1 tumors with nodal metastases has been associated with early metastatic features, highlighting the potential role of spatial miRNA variation in disease progression [51]. These spatial expression patterns correlate with tumor invasion and metastatic potential; however, while many observations remain correlative, emerging studies incorporating protein‐level validation provide increasing mechanistic support for these associations. These region‐specific differences in miRNA expression are likely shaped by local microenvironmental conditions, such as hypoxia, stromal composition, and immune cell infiltration, which in turn can drive spatially restricted activation of pathways governing invasion, survival, and immune evasion. However, interpreting apparent spatial miRNA gradients requires careful attention to several practical constraints. Multi‐region sampling design must ensure adequate geographic representation of the tumor, as undersampling of the invasive front or hypoxic core can yield misleading conclusions. Tumor purity is a critical confounder, since varying proportions of stromal and immune cells across sampled regions can drive apparent miRNA differences that reflect cellular composition rather than true tumor‐intrinsic gradients. Protein‐level validation by immunohistochemistry (IHC) of putative miRNA targets provides an important orthogonal measure for distinguishing biologically robust spatial signals from sampling artifacts. What constitutes a robust spatial signal should be defined prospectively: consistency across multiple regions, concordance between miRNA expression and target protein levels, and reproducibility across independent cohorts are minimum criteria for confident spatial inference. Importantly, the functional significance of these oncomiRNAs is supported by protein‐level validation of their downstream targets in human tumor specimens. For example, elevated miR‐21 expression has been consistently associated with reduced PTEN and PDCD4 protein expression levels by immunohistochemistry across multiple cancer types, linking it to activation of the PI3K/AKT pathway and enhanced tumor cell survival. Similarly, miR‐10b‐mediated suppression of HOXD10 has been demonstrated in invasive tumor regions, facilitating metastatic progression through derepression of pro‐migratory pathways, including RHOC signaling. In the immune and stromal context, miR‐155 has been shown to suppress targets including SOCS1 and SHIP1, thereby modulating cytokine signaling and immune cell activation states within the tumor microenvironment. The miR‐17‐92 cluster further contributes to proliferative signaling through regulation of targets such as PTEN, E2F1, and BIM, although spatial validation in human tissues remains comparatively limited. Collectively, these observations provide mechanistic evidence linking spatial oncomiRNA expression to downstream protein‐level alterations that drive region‐specific tumor behavior, as schematically illustrated in Figure 2A. Figure 2A depicts three spatially distinct tumor compartments, each characterized by a dominant oncomiRNA signature. In the hypoxic core, low oxygen tension stabilizes HIF‐1α, which drives upregulation of miR‐21, in turn suppressing PTEN and activating the PI3K/AKT survival pathway to promote resistance to apoptosis under nutrient‐deprived conditions. The proliferative zone is characterized by elevated miR‐17‐92 cluster expression, which promotes cell cycle progression through suppression of E2F1 feedback inhibitors and BIM, sustaining rapid tumor cell division. At the invasive margin, miR‐10b‐5p is upregulated, repressing HOXD10 and enabling RHOC‐driven cytoskeletal reorganization and epithelial–mesenchymal transition (EMT), facilitating local invasion and metastatic seeding. Collectively, these spatially partitioned oncomiRNA programs illustrate how region‐specific molecular signals converge to generate phenotypically distinct tumor subpopulations within a single tumor mass.

### 2.2 Temporal Heterogeneity of oncomiRNAs

While spatial heterogeneity emphasizes differences across tumor regions and metastatic sites, temporal heterogeneity reflects how oncomiRNA profiles evolve within individual patients over time, particularly in response to therapeutic interventions. These dynamic changes contribute to disease evolution and may influence treatment resistance and recurrence.

Longitudinal studies further support the dynamic regulation of oncomiRNAs during treatment. This includes both systemic therapies and liver‐directed interventions such as transarterial chemoembolization (TACE) and thermal ablation (TA), where treatment‐specific temporal miRNA changes have been observed. For instance, a prospective multicenter study in PDAC patients undergoing FOLFIRINOX chemotherapy demonstrated the temporal predictive value of circulating miRNAs. Serial serum analyses revealed that elevated baseline miR‐373‐3p and reduced miR‐194‐5p after one treatment cycle were independently associated with early tumor progression (miR‐373‐3p: OR 3.99, 95% CI 1.10–14.49; miR‐194‐5p: OR 0.91, 95% CI 0.83–0.99), but not with overall survival. These observations support the potential of dynamic miRNA profiling to identify early chemoresistance in PDAC [52]. Similarly, in lung adenocarcinoma, temporal profiling of plasma‐derived exosomal miRNAs before and after surgical resection revealed substantial shifts in miRNA expression, with 38 miRNAs upregulated and 37 downregulated in patients compared to healthy controls. Several upregulated miRNAs were also elevated in TCGA datasets and associated with overall survival. Notably, exosomal miR‐484 was significantly increased in patients and markedly decreased following tumor resection, highlighting its potential as a dynamic, noninvasive biomarker for disease monitoring [53].

In breast cancer, multiple longitudinal studies demonstrate dynamic changes in circulating miRNAs during therapy. For example, circulating miR‐155 levels are significantly elevated in patients compared to healthy controls (median 18.49 vs. 1.28, *p* < 0.0001) and decreased following surgery and chemotherapy (median 18.49 to 1.32, *p* < 0.0001), with associations to progression‐free survival (*p* = 0.038) [54]. Similarly, treatment‐associated changes in miR‐21 and miR‐221 have been reported during neoadjuvant chemotherapy (NACT) [55], while temporal patterns of miR‐125b and miR‐21 in stage II/III breast cancer patients correlate with treatment response and disease‐free survival [56].

An illustrative example of temporal heterogeneity in circulating miRNA expression was reported in metastatic testicular germ cell cancer (TGCC), where a panel of serum miRNAs was monitored at the onset of chemotherapy and throughout follow‐up, revealing dynamic changes over the course of treatment. These miRNAs were elevated at baseline, correlated with LDH (lactate dehydrogenase), and were significantly higher in patients who later relapsed or had refractory disease. Importantly, in patients achieving complete responses, miRNA levels declined sharply within the initial week of chemotherapy and remained suppressed during long‐term follow‐up. This temporal pattern underscores the utility of serial miRNA measurements for early relapse prediction, real‐time monitoring of therapeutic efficacy, and long‐term disease surveillance in TGCC [57].

Mechanistically, these temporal shifts likely reflect therapy‐induced selective pressures and adaptive tumor evolution, whereby resistant cellular subpopulations with distinct miRNA profiles are enriched over time. The miR‐17‐92 cluster, for example, has been implicated in promoting proliferation and resistance to apoptosis in multiple cancers, consistent with its proposed role in adaptive tumor persistence. Such dynamic miRNA reprogramming can modulate key pathways involved in apoptosis, PI3K/AKT‐mediated survival signaling, epithelial–mesenchymal transition, and immune evasion, thereby contributing to treatment resistance and disease recurrence.

Collectively, these findings underscore the potential of circulating miRNAs as temporal biomarkers for monitoring disease progression and therapeutic response. Despite these promising observations, interpretation of circulating miRNA dynamics remains technically challenging and highly dependent on pre‐analytical and analytical variables. Robust longitudinal profiling requires consistency in sample type (plasma versus serum), as differences in coagulation can influence miRNA abundance. Routine assessment of hemolysis (miR‐451/miR‐23a ratio) is essential, since even minimal red blood cell contamination can artificially alter circulating miRNA levels. Standardized protocols for exosome isolation and characterization, the use of exogenous spike‐in controls to monitor technical variability [58, 59, 60], and appropriate normalization strategies (such as global mean normalization or validated reference miRNA panels) are critical for ensuring comparability across time points [61]. In multicenter studies, harmonization of sample collection, processing, storage, and analytical workflows is essential for reproducibility. Failure to control these variables may lead to misinterpretation of temporal changes, where apparent shifts reflect technical artifacts rather than true biological dynamics [62]. Therefore, reported miRNA trajectories should be interpreted cautiously and, where possible, supported by complementary functional or pharmacodynamic validation. Beyond circulating miRNA variables, pseudo‐heterogeneity can also be introduced in tissue‐based multi‐region sampling studies. Differences in cold ischemia time between sampled regions can result in differential RNA degradation, generating apparent expression differences that are technical rather than biological in origin. Variable RNA degradation rates across tissue blocks, influenced by tissue thickness, fixation protocols, and processing delays, further compound this issue. In multicenter designs, batch effects arising from differences in RNA extraction platforms, amplification protocols, and sequencing runs must be explicitly addressed through appropriate batch correction strategies. Collectively, these technical confounders can create pseudo‐heterogeneity that mimics true biological spatial variation, underscoring the importance of rigorous quality control measures in spatiotemporal miRNA studies.

Together, these findings highlight the clinical value of monitoring temporal shifts in oncomiRNA expression to improve early detection of progression, assess treatment response in real time, and enable more precise, stage‐adapted, and dynamically responsive therapeutic strategies. However, many observations remain associative, and integration with functional validation studies and standardized methodologies will be essential to translate these findings into clinical applications. These temporal dynamics, exemplified by representative oncomiRNAs such as miR‐21, miR‐17‐92, and miR‐10b‐5p, are schematically illustrated in Figure 2B. Figure 2B presents a longitudinal trajectory across four treatment phases. At baseline, miR‐21 is elevated and supports tumor cell survival through PTEN suppression and PI3K/AKT activation, while also promoting immune evasion. During early treatment response, effective cytoreduction leads to suppression of miR‐21 levels in conjunction with declining tumor burden, providing an early pharmacodynamic readout of treatment activity. At the minimal residual disease (MRD) stage, therapy‐induced selective pressure enriches for drug‐tolerant persister cell subpopulations, characterized by upregulation of the miR‐17‐92 cluster, which drives adaptive proliferation and confers resistance to apoptotic stimuli. At relapse, miR‐10b‐5p becomes the dominant oncomiRNA, driving EMT‐associated metastatic dissemination and immune escape via suppression of HOXD10 and activation of RHOC signaling. The figure underscores that each of these phases represents a distinct therapeutic window, and that anti‐miR strategies targeting the appropriate oncomiRNA at the appropriate stage may offer context‐specific clinical benefit.

### 2.3 Stage‐Associated Heterogeneity of oncomiRNAs

Stage‐associated heterogeneity refers to variations in oncomiRNA expression across different stages or grades of disease, typically derived from cross‐sectional comparisons between patient groups rather than longitudinal analyses within the same individual. Multiple studies have demonstrated stage‐dependent alterations in oncomiRNA expression during tumor progression.

For instance, miR‐10b expression has been evaluated across increasing grades of cervical pathology—from cervicitis to LSIL, HSIL, and early‐stage cervical cancer—revealing a progressive decrease in expression. This downregulation was significantly associated with higher lesion grade, increased glandular involvement, and elevated p16 expression, suggesting a potential role in early disease progression and lesion stratification [63].

In contrast, gliomas exhibit an opposite trend, where miR‐10b is downregulated in low‐grade tumors but markedly upregulated in glioblastoma multiforme (GBM). This grade‐dependent increase indicates a context‐specific role in promoting tumor aggressiveness and malignant transformation [64], underscoring the tissue‐dependent nature of miRNA regulation.

Similarly, in colorectal cancer (CRC), stage‐ and metastasis‐associated differences in miRNA expression have been reported. Comparative analyses between primary tumors and matched liver metastases showed downregulation of miR‐28‐5p, miR‐143, and miR‐10b, alongside upregulation of miR‐122 and miR‐885‐5p in metastatic tissues. These patterns suggest dynamic reprogramming of miRNA expression during tumor dissemination, although the causative drivers of these alterations remain to be fully elucidated [65].

Mechanistically, stage‐associated changes in oncomiRNA expression likely reflect the cumulative impact of genetic and epigenetic alterations acquired during tumor evolution, in conjunction with dynamic interactions within the tumor microenvironment. Early‐stage lesions tend to exhibit miRNA profiles linked to proliferation control and immune surveillance, whereas advanced tumors display signatures that facilitate invasion, epithelial–mesenchymal transition, metastasis, and immune evasion. These transitions highlight the functional contribution of miRNAs to phenotypic shifts across disease stages.

Table 1 summarizes miRNAs exhibiting spatial, temporal, and stage‐specific dysregulation across multiple malignancies, drawing from both cross‐sectional investigations and indirect longitudinal analyses. It is important to acknowledge a significant limitation of the body of evidence represented in Table 1: the majority of studies are single‐center, small‐sample, and retrospective in design, which substantially limits their clinical generalizability. The absence of multicenter prospective validation means that many of the reported associations should be considered hypothesis‐generating rather than clinically actionable. Future studies should prioritize multicenter, prospective, longitudinal cohort designs to validate these findings across diverse patient populations, tumor types, and treatment settings. Collectively, stage‐associated heterogeneity in oncomiRNA expression represents a dynamic layer of molecular reprogramming during tumor progression. While these patterns offer promising avenues for stage‐specific biomarker development and therapeutic stratification, their variability across tumor types emphasizes the need for context‐specific validation. Together, spatial, temporal, and stage‐associated heterogeneity constitute complementary dimensions of oncomiRNA regulation that shape tumor evolution and influence therapeutic response.

**TABLE 1:** Summary of oncomiRNAs exhibiting spatial, temporal, and clinical heterogeneity across human cancers and their translational relevance.

Spatial heterogeneity								
Cancer type	Context	Dysregulated miRNAs	Assay	Sample size	Direction	Clinical relevance	Evidence tier	Refs.
Breast cancer	Multi‐region tumor + lymph node metastases	miR‐10b, ‐210, ‐31, ‐335	RT‐qPCR	*n* = 16 (multi‐region sampling; 132 total samples)	↕ (spatially heterogeneous)	Significant intratumoral and metastatic heterogeneity (CV ~40%), indicating potential sampling bias	Tissue, multi‐region	[50]
Breast cancer	Tumor vs. normal region	miRNA‐21, ‐200b, ‐200a, ‐191	RT‐qPCR	*n* = 33	↕ (region‐dependent variation)	Consistently distinguishes tumor from normal tissue, but expression varies across intratumoral regions	Tissue, multi‐region	[66]
Colorectal cancer	Tissue (invasive front of pT1 CRCs with nodal metastases)	miR‐17‐3p, ‐92a	Microarray	*n* = 56	**↑** in LNM‐positive tumors	Region‐specific upregulation at invasive front associated with LNM	Tissue, multi‐region	[51]

Temporal heterogeneity								
Cancer type	Context	Dysregulated miRNAs	Assay	Sample size	Direction	Clinical relevance	Evidence tier	Refs.
HCC and CRC liver metastases	Plasma (multiple time points; TA vs. TACE)	miR‐122 (liver injury) and ‐200a (EMT‐related)	RT‐qPCR	*n* = 20 (10 TACE, 10 TA)	↔ (time‐ and treatment‐dependent)	TA induces immediate miRNA elevation, whereas TACE shows delayed increase (24 h), highlighting treatment‐specific temporal regulation and potential for therapy monitoring	Circulating, longitudinal	[67]
NSCLC (EGFR‐mutated)	Plasma (EGFR‐TKI treatment, longitudinal)	miR‐21	RT‐qPCR	*n* = 39	↔ (time‐dependent increase in SD)	Temporal changes in circulating miR‐21 correlate with EGFR‐TKI response, with increased levels at 2 months associated with stable disease	Circulating, longitudinal	[68]
Breast cancer	Tissue (pre‐ vs. post‐NACT)	miR‐124a, ‐137	RT‐qPCR	*n* = 34	↑/↓ (HR‐dependent)	Post‐NACT changes in miR‐124a and miR‐137 are associated with hormone receptor status but not with treatment response	Tissue, paired longitudinal	[69]
Lung adenocarcinoma	Plasma (exosomal miRNA; pre‐ vs. post‐surgery)	miR‐151a‐5p, ‐10b‐5p, ‐192‐5p, ‐106b‐3p, and ‐484	Exosomal RNA‐seq + RT‐qPCR validation	*n* = 6 cases + 6 controls	↑ in cancer; miR‐484 ↓ post‐surgery	Exosomal miRNAs identified as potential prognostic biomarkers; miR‐484 shows dynamic reduction after tumor removal	Circulating, longitudinal	[53]

Clinical heterogeneity								
Cancer type	Context	Dysregulated miRNAs	Assay	Sample size	Direction	Clinical relevance	Evidence tier	Refs.
Breast cancer	FFPE tissue	miR‐205, ‐21	RT‐qPCR	*n* = 84 cases + 13 controls	↑ (associated with poor prognosis)	miR‐21 associated with shorter DFS; miR‐205 associated with shorter DFS and OS, indicating higher relapse risk	Tissue, associative	[70]
Prostate cancer (PCa)	Plasma + tissue (high vs. low grade); PCa vs. BPH (discovery + validation cohorts)	miR‐373‐3p	RT‐qPCR	Discovery: *n* = 3 (high grade) + 3 (low grade) + 3 BPH; validation: *n* = 42 PCa + 42 BPH	**↑**	Overexpressed in high‐grade PCa; associated with KPNA2 downregulation and tumor grade stratification	Circulating + tissue, associative	[71]
Colorectal cancer	Tissue (tumor vs. adjacent; clinicopathological correlation)	miR‐17‐5p	RT‐qPCR	*n* = 30	**↑**	Upregulated in tumors and associated with stage, lymph node metastasis, and enhanced proliferation, invasion, and survival	Tissue, associative	[72]
Breast cancer	Stratifies LNM‐positive vs. LNM‐negative patients	miR‐10b, ‐373	RT‐qPCR	*n* = 60 cases + 10 controls	**↑** in LNM+	Circulating miR‐10b and miR‐373 distinguish LNM‐positive from LNM‐negative patients with good diagnostic accuracy	Circulating, associative	[73]

Abbreviations: BPH, Benign prostatic hyperplasia; CRC, colorectal cancer; CV, coefficient of variation; DFS, disease‐free survival; EGFR‐TKI, epidermal growth factor receptor‐tyrosine kinase inhibitor; EMT, epithelial–mesenchymal transition; FFPE, formalin fixed paraffin embedded; KPNA2 gene, karyopherin subunit alpha 2; LNM, lymph node metastasis; NACT, neoadjuvant chemotherapy; RT‐qPCR, reverse transcription quantitative PCR; SD, stable disease; TA, thermal ablation; TACE, transarterial chemoembolization.

### 2.4 Significance of Spatiotemporal Regulation in Therapeutic Interventions

miRNAs possess the capacity to modulate complex molecular networks by simultaneously regulating multiple target genes, highlighting their therapeutic potential in cancer. Therapeutic strategies broadly involve either restoration of tumor‐suppressive miRNAs using synthetic mimics or inhibition of oncogenic miRNAs using antisense‐based approaches. Replacement strategies include engineered miRNA agonists delivered via viral vectors or plasmid‐based systems, whereas inhibitory approaches utilize antisense oligonucleotides (ASOs), locked nucleic acids (LNAs), chemically modified oligonucleotides, CRISPR/Cas‐based systems, small molecule inhibitors, or miRNA sponges. These approaches enable bidirectional modulation of miRNA activity for therapeutic benefit.

Evidence supporting the clinical feasibility of miRNA‐targeted therapy is provided by a first‐in‐human phase I trial of LNA‐i‐miR‐221, a 13‐nucleotide locked nucleic acid antisense oligonucleotide targeting miR‐221. In this dose‐escalation study involving patients with advanced solid tumors, treatment was administered via short intravenous infusions over four consecutive days and was well tolerated, with no grades 3–4 toxicities. Preliminary clinical activity was observed, including disease stabilization in approximately 50% of patients and a partial response in colorectal cancer. Pharmacodynamic analyses demonstrated dose‐dependent reductions in circulating and, where available, tissue miR‐221 levels, accompanied by increased expression of downstream targets such as CDKN1B/p27 and PTEN. Although not derived from longitudinal patient‐specific profiling, the incorporation of temporal pharmacodynamic monitoring highlights the feasibility of controlled, time‐dependent modulation of miRNA activity in a clinical setting [74].

The clinical relevance of miRNA dysregulation is further supported in multiple myeloma, where reduced expression of the miR‐221/222 cluster has been associated with advanced disease stages. This downregulation, particularly in R‐ISS stage III patients, correlates with early disease progression and inferior overall survival. Integration of miR‐221/222 expression into existing risk stratification models has been shown to improve prognostic accuracy and identify patients at risk of suboptimal response to first‐line therapy, supporting its potential role in precision oncology [75].

Despite these advances, the successful translation of miRNA therapeutics remains constrained by challenges related to delivery, biodistribution, and safety. Various delivery platforms, including lipid nanoparticles (LNPs), GalNAc‐conjugated systems, and exosome‐mimetic vesicles, are being developed to enhance tumor‐specific uptake [76] (Table 2). However, achieving efficient intratumoral delivery while minimizing systemic exposure continues to be a major limitation. A key challenge in miRNA therapeutics is the balance between systemic biodistribution and effective tumor penetration, as many delivery systems preferentially accumulate in the liver, limiting bioavailability at tumor sites. Furthermore, optimal dosing strategies must account for narrow therapeutic windows and potential immune activation. In addition, miRNA therapeutics may induce innate immune responses, particularly via Toll‐like receptor (TLR7/8) activation, and may exhibit off‐target effects due to partial sequence complementarity [91, 92]. Robust pharmacokinetic and pharmacodynamic (PK/PD) assessment is therefore essential, with emphasis on confirming target engagement through downstream protein modulation rather than relying solely on circulating miRNA levels [93]. Notably, alterations in plasma or serum miRNA levels may not accurately reflect intratumoral activity in the absence of tissue‐based validation.

**TABLE 2:** miRNA therapeutic platforms: delivery strategies and translational challenges.

Modality	Strategy	Advantages	Limitations	Example	Clinical maturity
miRNA mimics	Synthetic double‐stranded RNAs that restore tumor‐suppressor miRNA function	Restore lost miRNA activity; enable broad regulation of multiple oncogenic targets	Poor stability; rapid degradation; delivery challenges; off‐target effects	miR‐34 mimic (MRX34; clinical trial terminated due to immune toxicity) [77]	Clinical trial (terminated)
ASOs/LNAs	Single‐stranded antisense oligonucleotides that inhibit oncogenic miRNAs (e.g., miR‐221)	High specificity and stability (LNA chemistry); demonstrated anti‐tumor activity	Activity may depend on TP53 status; potential off‐target and immune‐related effects; delivery challenges	LNA‐i‐miR‐221 restores TP53 pathway regulators (e.g., TP53BP2, TP53INP1) and induces apoptosis [78, 79]	Early clinical (phase I)
miRNA sponges/decoys	Competitive inhibitors with multiple miRNA binding sites that sequester miRNAs and prevent target interaction	Inhibit entire miRNA families; tunable design; enables stable and long‐term inhibition	Require sustained expression; risk of saturating endogenous miRNA machinery; delivery and scalability challenges; limited clinical translation	miR‐15a/16 sponge (tumor models) [80]; miR‐133 sponge (cardiac hypertrophy) [81]; miR‐326 sponge (autoimmune model) [82]	Preclinical
CRISPR/Cas‐based editing (including dCas9)	Genome editing or transcriptional modulation of miRNA genes (e.g., miR‐155)	Precise and programmable targeting; potentially durable effects; enables modulation of oncogenic miRNAs	Off‐target effects; delivery challenges; safety concerns; limited clinical translation	CRISPR/dCas9‐mediated targeting of miR‐155 in liver cancer models using lentiviral and exosome‐based delivery [83, 84]	Preclinical
LNPs	Lipid based nanoparticles encapsulating miRNA mimics/ASOs for systemic delivery	Efficient cellular uptake; enable functional delivery; clinically validated in nucleic acid therapies	Liver accumulation; limited tumor penetration; potential toxicity	LNP‐mediated delivery of miR‐634 suppresses tumor growth in pancreatic cancer models; widely used in siRNA therapeutics (including FDA‐approved platforms) [85]	Preclinical; platform clinically validated
GalNAc‐conjugated systems	Ligand‐mediated delivery targeting hepatocytes via ASGPR	High liver specificity; efficient receptor‐mediated uptake; improved pharmacokinetics; clinically validated RNAi platform	Limited to liver‐targeted diseases; not suitable for most solid tumors	GalNAc–siRNA conjugates with demonstrated clinical advancement (multiple Phase III trials) [86, 87]	Clinical trial (phase III)
Exosome‐based delivery	Natural vesicles used to transport miRNAs between cells	High biocompatibility; low immunogenicity; protect miRNAs from degradation; enable targeted delivery	Challenges in isolation, standardization, scalability, and cargo loading; potential off‐target effects	MSC‐derived exosomes loaded with therapeutic miRNAs inhibit tumor growth and enhance therapy response in preclinical models [88]	Preclinical
Viral vectors (lentivirus, AAV)	Gene delivery systems expressing miRNA mimics or inhibitors	Long‐term expression; high transduction efficiency; stable gene transfer	Immunogenicity; insertional mutagenesis risk; safety and regulatory challenges	Lentiviral and AAV‐mediated miRNA delivery in gene therapy applications with sustained expression in preclinical and early clinical studies [89, 90]	Preclinical/early clinical

Abbreviations: AAV, adeno‐associated virus; ASGPR, asialoglycoprotein receptor; ASO, antisense oligonucleotide; LNA, locked nucleic acid; LNP, lipid nanoparticle; MSC, mesenchymal stem cell; TLR, toll‐like receptor.

Understanding the spatiotemporal dynamics of miRNA expression is critical for optimizing therapeutic interventions. miRNA expression patterns often vary across both disease stages and anatomical compartments, implying that effective therapeutic strategies may require stage‐specific and context‐dependent modulation. Longitudinal profiling approaches can help identify temporal windows of vulnerability, enabling optimal timing of intervention, improved patient stratification, and real‐time monitoring of therapeutic response. Furthermore, advances in targeted delivery systems may facilitate spatially restricted modulation of miRNAs within tumor tissues, thereby reducing off‐target toxicity.

Collectively, integrating spatiotemporal insights into miRNA biology provides a framework for the rational design of therapeutic strategies that are both biologically informed and clinically adaptable. Such approaches have the potential to enhance treatment efficacy, limit adverse effects, and improve outcomes in patients with heterogeneous malignancies. In extending the translational scope of this framework, it is noteworthy that complementary molecular pathways are increasingly being explored in conjunction with miRNA‐based strategies. For instance, the SUMOylation pathway has been investigated in the context of preclinical models combining SUMOylation inhibitors with immune checkpoint inhibitors, offering insights into how post‐translational regulatory mechanisms interact with the tumor immune microenvironment and may synergize with miRNA‐targeted approaches to achieve more durable anti‐tumor responses [94]. Furthermore, the molecular mechanisms by which gut microbiota metabolic reprogramming, particularly through regulation of the bile acid‐FXR axis, govern lipid metabolism reprogramming in the host represent an emerging dimension of the tumor metabolic landscape [95]. Incorporating these regulatory networks into mechanistic interpretations of oncomiRNA function may enrich the systems‐level understanding of how miRNA dysregulation integrates with metabolic and immune reprogramming in cancer progression.

## 3 Conclusions and Future Directions

Future research should prioritize systematic investigation of the spatiotemporal dynamics of oncomiRNA expression through integrated multi‐region and longitudinal sampling strategies, complemented by advanced spatial omics and liquid biopsy technologies. Such approaches will be essential for identifying context‐specific molecular vulnerabilities and defining optimal therapeutic windows for intervention.

A key translational priority is the development of adaptive therapeutic frameworks incorporating real‐time miRNA monitoring. As a hypothetical illustration (thresholds not yet validated in prospective trials), early on‐treatment (e.g., Cycle 1, Day 15) corresponding to an initial pharmacodynamic assessment window after first drug exposure, circulating oncomiRNAs could be evaluated to guide therapeutic decisions, where predefined increases in markers such as miR‐21 or miR‐10b (e.g., ≥ 2‐fold from baseline) trigger the addition of anti‐miR‐based interventions. Conversely, lack of a composite response, defined by persistent oncomiRNA elevation alongside ctDNA positivity, may serve as a futility signal, prompting treatment modification or escalation. These are presented explicitly as unvalidated, exploratory response‐adaptive strategies to illustrate how spatiotemporal miRNA dynamics might eventually be translated into clinically actionable decision frameworks, pending prospective validation.

Among emerging applications, circulating miRNA‐based surveillance in testicular germ cell tumors and minimal residual disease (MRD) detection in colorectal cancer represent particularly promising candidates for near‐term clinical translation, given their pronounced temporal dynamics and increasing clinical validation. In these contexts, dynamic changes in miRNA levels relative to baseline may provide early indicators of relapse or residual disease, especially when integrated with complementary biomarkers such as ctDNA. Predefined thresholds (e.g., a ≥ 2‐fold increase from post‐treatment nadir or persistent failure to normalize to baseline levels) could be prospectively evaluated to standardize interpretation of these temporal changes. Similarly, peri‐operative monitoring in lung adenocarcinoma represents a potential application, where post‐surgical declines followed by re‐elevation of specific exosomal miRNAs (e.g., miR‐484) may indicate early recurrence, although standardized thresholds remain to be established.

Advances in delivery technologies remain critical for successful clinical translation. Platforms such as lipid nanoparticles, conjugate‐based systems, and extracellular vesicles offer promising strategies for targeted delivery; however, challenges related to biodistribution, tumor penetration, immune activation, and off‐target effects persist. Achieving spatially precise and temporally controlled miRNA modulation remains a central limitation. Furthermore, circulating miRNA changes should be interpreted in conjunction with pharmacodynamic evidence of intratumoral target engagement to ensure accurate assessment of therapeutic efficacy. Optimization of dosing strategies, including route of administration, dosing frequency, and escalation schedules will also be necessary, as variability in tissue uptake and duration of pharmacodynamic effects complicates standardization.

Finally, integration of artificial intelligence approaches offers substantial potential to operationalize spatiotemporal miRNA dynamics in precision oncology. Future models should incorporate multimodal feature sets, including spatially resolved miRNA expression patterns, radiomic features, and clinicopathological variables such as stage, grade, and treatment history. These inputs can be integrated within machine learning frameworks to generate dynamic risk scores for progression or relapse, enable early prediction of therapeutic response, and support adaptive treatment decisions, including decision support for initiation of miRNA‐targeted add‐on therapies. Model performance should be rigorously evaluated using time‐dependent AUC, calibration metrics, and measures of clinical utility such as decision curve analysis.

Collectively, these advances position spatiotemporal miRNA profiling as a promising component of next‐generation precision oncology. However, their successful implementation will depend on prospective validation, assay standardization, and integration into clinically feasible workflows.

## Author Contributions

**T. Jeethy Ram:** conceptualization, writing – original draft. **Muralee Damodaran:** conceptualization, writing – review and editing.

## Funding

The authors have nothing to report.

## Ethics Statement

The authors have nothing to report.

## Conflicts of Interest

The authors declare no conflicts of interest.

## Data Availability

Data sharing not applicable to this article as no datasets were generated or analyzed during the current study.
